# Volumetric Modulated Arc Therapy Capabilities for Treating Lower-Extremity Skin Affected by Several Merkel Cell Carcinoma Nodules: When Technological Advances Effectively Achieve the Palliative Therapeutic Goal while Minimising the Risk of Potential Toxicities

**DOI:** 10.3390/medicina57121379

**Published:** 2021-12-18

**Authors:** Gianluca Ferini, Vito Valenti, Ivana Puliafito, Salvatore Ivan Illari, Valentina Anna Marchese, Giuseppina Rita Borzì

**Affiliations:** 1 REM Radioterapia srl, Via Penninazzo 11, I-95029 Viagrande, CT, Italy; vito.valenti@grupposamed.com (V.V.); valentina.marchese@grupposamed.com (V.A.M.); 2 Medical Oncology Unit, Mediterranean Institute of Oncology, I-95029 Viagrande, CT, Italy; ivana.puliafito@grupposamed.com; 3 Fondazione Istituto Oncologico del Mediterraneo, I-95029 Viagrande, CT, Italy; salvatore.illari@fondazioneiom.it; 4 Humanitas C.C.O., I-95125 Catania, CT, Italy; giuseppina.borzi@grupposamed.com

**Keywords:** volumetric modulated arc therapy, extended field radiotherapy, merkel cell carcinoma, skin cancer, radiotherapy for lower extremity tumors, lymphedema, bone sparing, junction of radiation fields’ management, radiation adverse events, quality of life

## Abstract

The peculiar and rare clinical condition below clearly requires a customized care approach in the context of personalized medicine. An 80-year-old female patient who was subjected in 2018 to surgical removal of a cutaneous Merkel cell carcinoma (MCC) nodule located on the posterior surface of the left thigh and to three subsequent palliative radiotherapy treatments developed a fourth relapse in October 2020, with fifteen nodular metastases located in the left thigh and leg. Since the overall macroscopic disease was still exclusively regionally located and microscopic spread was likely extended also to clinically negative skin of the thigh and leg, we performed an irradiation of the whole left lower extremity. For this purpose the total target (65.5 cm) was divided into three sub-volumes. Dose prescription was 30 Gy in 15 daily fractions. A sequential boost of 10 Gy in 5 daily fractions was planned for macroscopic nodules. Plans were calculated by means of volumetric modulated arc therapy (VMAT) with the field overlap technique. Thanks to this, we obtained a homogeneous dose distribution in the field junction region; avoidance structures were delineated in the central part of the thigh and leg with the aim of achieving an optimal superficial dose painting and to reduce bone exposure to radiation. This case study demonstrates that VMAT allows for a good dose coverage for circumferential cutaneous targets while sparing deeper organs at risk. A reproducible image-guided set-up is fundamental for an accurate and safe dose delivery. However, local treatments such as radiotherapy for very advanced MCC of the lower extremities might have limited impact due to the high probability of systemic progression, as illustrated in this case. Radiation is confirmed as being effective in preventing MCC nodule progression toward skin wounding.

## 1. Introduction

Among oncologic outcomes, prolonging local control (LC) is particularly important, at least as much as improving survival chances. This is also true in palliative settings where a symptomatic progression of a local disease can adversely affect patients’ quality of life. For example, cutaneous Merkel cell carcinoma (MCC) nodules may occasionally evolve from painless dome-shaped violaceous papules to ulceration, which could be massive in case of large lesions [1]. This particular type of skin lesion, precisely because it develops within a microenvironment contaminated by cancer cells, could be difficult to manage [2]. While ulceration is an uncommon feature, cutaneous/subcutaneous neoplastic diffusion occurs more frequently. In fact, MCC cells have a remarkable tropism for intradermal lymphatics [3]. In case of tumor location in the extremities, the possible subsequent stop of lymph flow can cause significant and dysfunctional swelling of the arm or leg. In the latter case, a severe deambulation impairment may develop, along with serious psychological distress [4]. Moreover, both ulceration and lymphedema predispose patients to dangerous infections [5]. For these reasons, the deleterious effects of local disease progression need to be mandatorily prevented [6]. Merkel cell carcinomas (MCCs) in the lower extremities have a very poor prognosis, with 14% of cases having a 5-year survival rate [7]. The relative rarity of MCC and the lack of prospective key trials for palliative therapeutic options contribute to the difficulty in defining an effective therapeutic proposal in such a disease scenario. New immunotherapies such as pembrolizumab, avelumab, nivolumab, ipilimumab are available but not always fully effective for changing disease prognosis [8]. However, MCC cells have proved to be fairly radiosensitive, and lower palliative radiation doses could be no worse than higher curative ones, at least in terms of local control in palliative settings [9,10,11]. Indeed, the natural course of this skin cancer is marked by more regional and distant relapses than local ones [12]. These facts could make radiation oncologists reconsider the usefulness of palliative extended field radiotherapy for locoregional control even in massive limb involvement. The wide extension and irregular shape of such a target make irradiation particularly challenging [13].

Here we present a clinical application of volumetric modulated arc therapy (VMAT) in the palliative treatment of the left lower limb of a patient affected by multiple dermal metastases from MCC, some of which were erythematosus but not yet ulcerated. The aim of this approach was to delay local disease progression toward related complications.

## 2. Case Study

**Figure 1:**
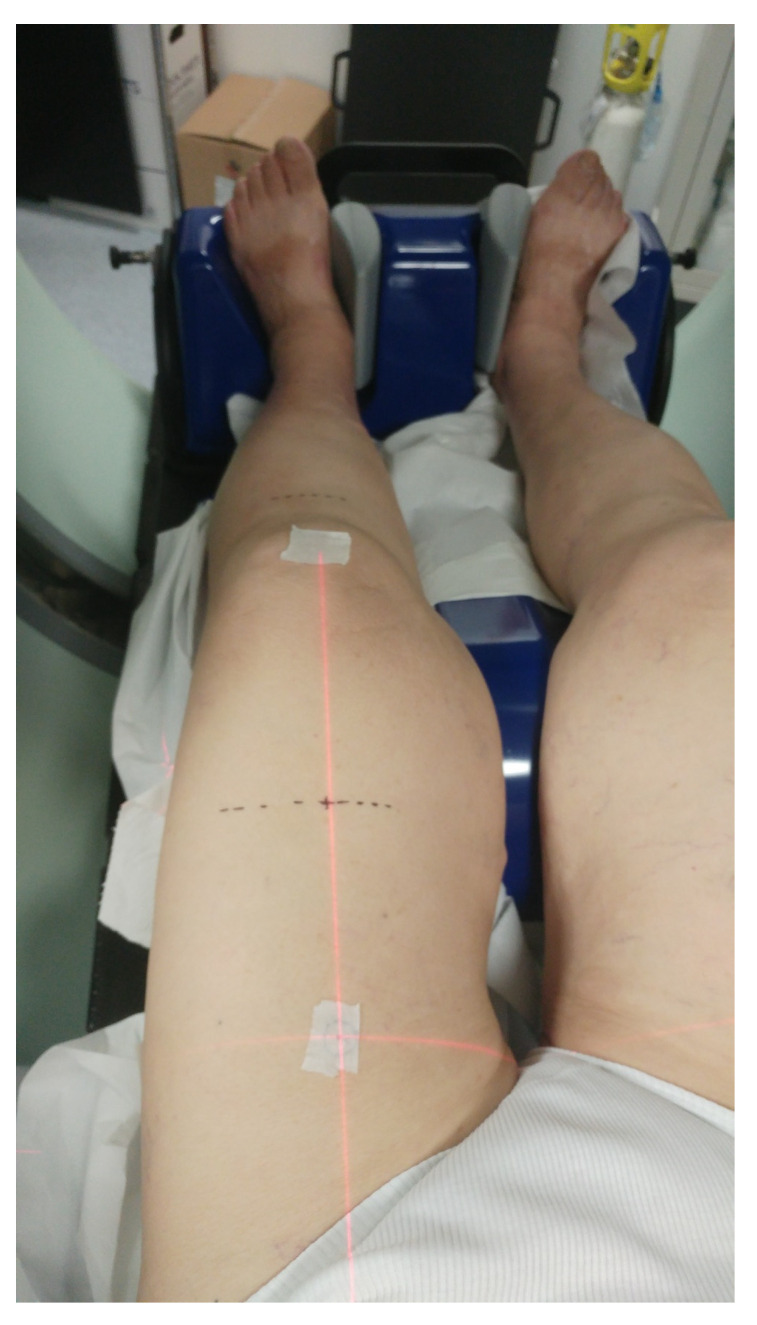
Patient positioning during CT simulation: legs and feet were immobilized by means of a ProSTEP device and landmarks were marked on the skin for isocenter determination.

**Figure 2:**
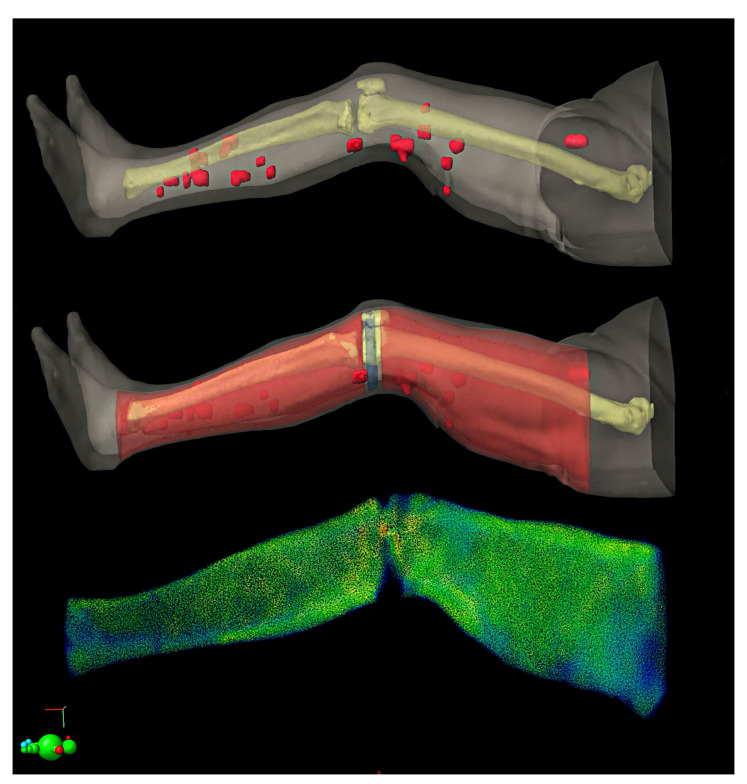
Three-dimensional reconstruction of macroscopic nodules (**top**), treatment volumes of the thigh and leg (red) (**middle**) and dose distribution (**bottom**).

In May 2018, an 80-year-old female patient with controlled diabetes and hypertension and no other relevant comorbidities was submitted to a surgical removal of a purplish painful cutaneous nodule of 4.3 cm located on the posterior surface of the left thigh, histologically positive for Merkel cell carcinoma. The patient was therefore a candidate for maintenance treatment with avelumab. Nevertheless, three subsequent limited relapses occurred: (1) the first, located in the popliteal fossa, in November 2019; (2) the second, on the midline skin of the anterior surface of the thigh and in the groin lymph nodes, in March 2020; (3) the third, on the medial distal surface of the thigh, in May 2020. Each of the three locations was treated with involved field radiotherapy with a palliative total dose of 30 Gy in 10 consecutive fractions of 3 Gy/day, leading to a lasting clinical complete local response without any side effects. The basically slow disease progression with limited tumor burden at every relapse ensured that there was no strict need to change systemic treatment. Besides, avelumab was very well tolerated by the patient. In October 2020, in the context of mild lower limb lymphedema (grade 1 according to the International Society of Lymphology (ISL) Staging System), the patient developed a fourth clinically- and 18F-FDG-PET-detected relapse. This consisted of fifteen moderately painful and itchy separate nodules in the lower left extremity, the latter being the only body site of clinical disease. Three wide clusters, located in the following regions, could be distinguished: (1) groin lymph nodes, more distal than the previous ones; (2) skin nodules on the bottom half of the posterolateral aspect of the thigh; and (3) skin nodules below the knee. Since the overall macroscopic disease was still regionally located and microscopic spread was likely extended even to clinically uninvolved skin of the thigh and leg, we proposed to the patient a whole left-limb irradiation with a palliative intent. However, with this approach, the retreated areas, i.e., those resulting from the overlap between the previous involved field irradiations and the last extended one, were very limited in size, so that they did not threaten the treatment tolerability. On the basis of a 3 mm-thick slice CT simulation (extended field of view = 820 mm) in the most suitable position on a PROSTEP™ device (Figure 1), we contoured the whole circular space between the body surface and the muscle and bone tissues of the leg as the planning target volume (PTV). We delineated the proximal edge of such a PTV at the level of the inguinal ligament, just below the previous radiation field (March 2020), and the distal one at the level of the medial malleolus. A PTV minus (≈3 cm longitudinally) was intentionally created to spare the popliteal lymph nodes while preserving an adequate dose coverage of the neighboring skin lesions. Femur and tibia were contoured as organs at risk (OAR) (see Figure 2, the two topmost images). The total length of the PTV was 65.5 cm. Dose prescription was 30 Gy in 15 daily fractions. A sequential boost of 10 Gy in 5 daily fractions was scheduled for macroscopic nodules.

Since the irradiation of such large volumes is impossible to plan with a single isocenter due to the well-known LINAC limitations, it is necessary to use multiple isocenters. Consequently, the field junctions are markedly influenced by uncertainties in the intrafraction set-up, which could cause strong overdoses and/or underdoses inside the PTV, and eventually important toxicities. In order to obtain results that are less sensitive to positional uncertainties and to successfully achieve an optimal management of the junction, the VMAT with the field overlap technique was used for the present treatment, similar to that developed for craniospinal irradiation [14,15]. In particular, the target was divided into three sub-volumes with an overlap length of 6 cm. The latter extent was arbitrarily chosen according to the literature data on field junction planning [14,15]. Each sub-volume had its own isocenter and was planned using two full VMAT arcs with collimator angles of 45°/315°, for a total of six VMAT arcs. The dimensions of the planning fields, starting from the thigh towards the feet, were the following: 30 cm × 27 cm, 28 cm × 24 cm and 27 cm × 18 cm for the first, second and third sub-volume, respectively. To simplify patient positioning during treatment, the three nearly equidistant isocenters were collinear (only the longitudinal coordinate changed). Moreover, to cover the entire PTV with the aim of delivering an adequate dose up to the skin surface and to save the bone, avoidance structures were delineated in the central part of the thigh and leg. Therefore, in a single plan, two full coplanar VMAT arcs were used for each of the three isocenters, minimizing the dose to the femur, tibia and popliteal fossa.

**Figure 3:**
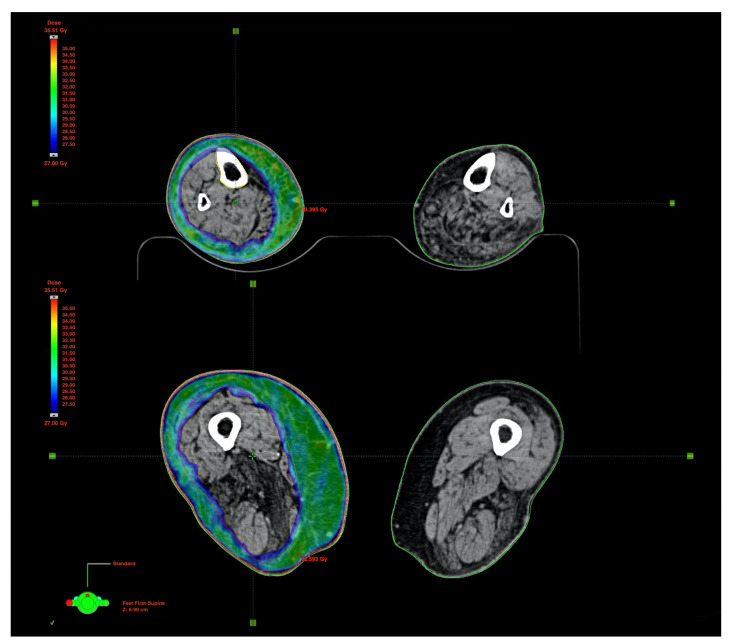
VMAT distribution curves of the 90% isodose covering PTV_leg (**top**) and PTV_thigh (**bottom**). VMAT, volumetric modulated arc therapy; PTV, planning target volume.

While for plan optimization three separate PTVs were considered, only two, one for the thigh, the other for the leg (PTV_thigh and PTV_leg as shown in Figure 2 and Figure 3) were evaluated for dose coverage. Treatment planning was performed on the Eclipse 13.6 treatment planning system (TPS) and the dose was computed using the analytical anisotropic algorithm (AAA) with 2.5 mm calculation grid resolution. Treatment was delivered by means of a Varian Trilogy linear accelerator (LINAC) equipped with a Millennium 120 multi-leaf collimator and an Exact IGRT couch (Varian Medical Systems, Palo Alto, CA, USA). Before treatment delivery, the patient was subjected to daily online image-guided radiotherapy (IGRT) using cone-beam computed tomography (CBCT), in order to reduce set-up uncertainty. Thanks to the use of the PROSTEP device, the set-up corrections were kept below 3 mm in all spatial directions. Indeed, the immobilization system used and the verification of the setup through CBCT are particularly suitable for this kind of patient, since the first allows for proper immobilization and stability of the lower limbs and the second for satisfactory assessment of the accuracy of the positioning based on bone landmarks and surrounding soft tissues.

**Figure 4:**
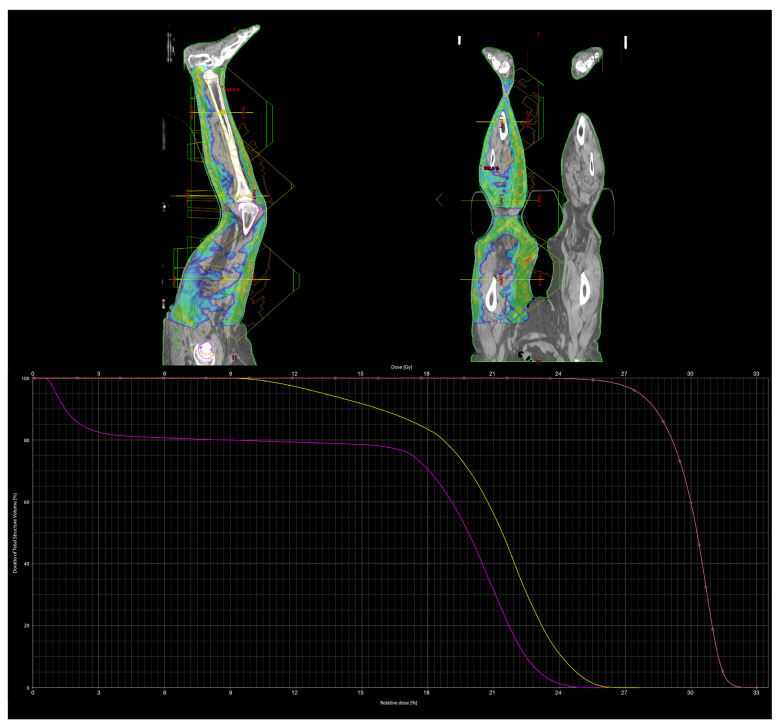
90% isodose lines in sagittal and coronal planes with fields and dose–volume histogram (DVH) for PTV (light red), femur (fuchsia) and tibia (yellow).

We searched for and obtained a dose to 95% of the target volume (D95%), equal to 90% of the prescription dose (Figure 3). This means that almost the whole target skin (95%) was approximately covered by a dose of at least 27 Gy (90%) (Figure 4). Hot spots were limited to 110% of the prescription dose. Mean doses to the femur and tibia were 16.6 Gy and 20.7 Gy, respectively. Such a substantial difference was due to a more superficial location of the tibia compared to the femur. The PTV contained the surface of the skin; despite this, the most superficial part of the target was not covered by 95% of the prescription dose due to the build-up effect typical of 6 MV photon beams. However, the bolus was not usable for this type of treatment because there is no bolus able to cover such a large surface and its positioning would not have been reproducible during daily treatment.

**Figure 5:**
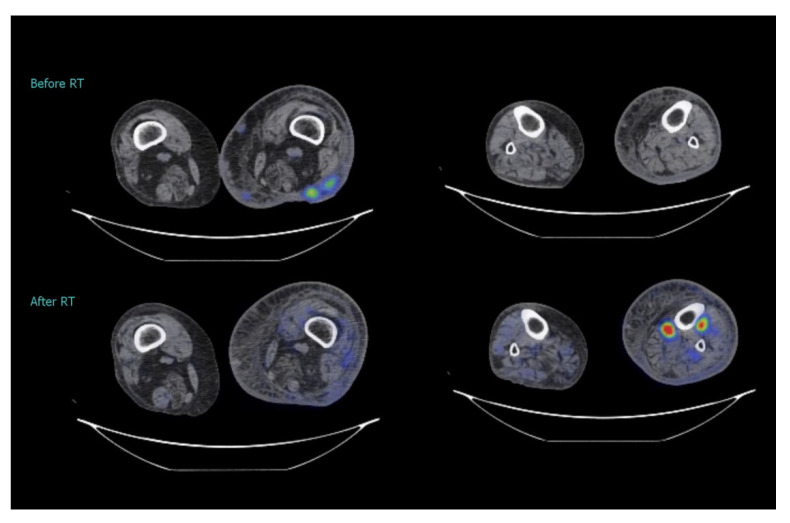
Comparison between 18F-FDG PET images: three months after radiotherapy several 18F-FDG-avid nodules appeared at the interface between muscles and bones. 18F-FDG PET, fluorodeoxyglucose positron emission tomography.

This radiotherapy treatment failed to prolong progression-free survival, as expected: three months after the end of therapy, an 18F-FDG positron emission tomography (PET) documented a broad lymph-node progression in mediastinal, epigastric, common and external iliac regions. As a consequence, the patient tumor disease never appeared in complete remission at the instrumental evaluation. Interestingly, no skin lesions, except one arising in the underdosed popliteal fossa (≈2 cm), were visible in the PET images, but several CT-hypodense FDG-avid nodules (not lymph nodes) were reported deeply in the interface between muscles and bones, where the mean radiation dose was approximately half the prescribed dose. However, the patient’s symptoms improved rapidly during the treatment and all irradiated nodules were clinically undetectable a month later (Figure 5). No acute RT-related toxicities were detected. Only five months later, the lower limb lymphedema worsened to grade 2 according to the ISL stage system [16], likely due to the increasingly massive involvement of iliac lymph nodes; no new skin lesions occurred, apart from the still-painless one previously detected in the underdosed popliteal fossa. Due to the large progression of disease, systemic treatment was switched to cisplatin plus pemetrexed, with poor results. The patient died 8 months later of acute respiratory failure, likely related to a large mediastinal tumor burden, in the context of an excellent in-field local control. The limited survival did not allow us to appreciate a lasting reduced risk of the femur and tibia fracture following a radiation exposure that was lower than that from radiotherapy techniques that are unable to spare leg bones, such as 3D-CRT.

## 3. Discussion

The reported case shows that even extremely complex targets can be irradiated thanks to more and more new performing radiotherapy techniques [17]. The dose delivery characteristics of VMAT allow for a superficial circumferential irradiation while sparing organs at risk centered within the target (femur and tibia in our case) by exploiting the tangential effect [13].

Since we were aware of the natural history of M1a MCC of the lower extremity [7], we decided to treat macroscopic nodules with a palliative dose of 40 Gy in 2 Gy/fraction/day. On the other hand, we lowered the dose to 30 Gy for the clinically uninvolved skin. The latter dose prescription was deemed useful by us to contrast tumor cell migration. In fact, two other critical issues led us not to exceed that dose: (i) the impact of radiation on lymphedema [18] and (ii) concerns about the risk of pathological fracture after radiotherapy. Accordingly, when treating extremity soft-tissue sarcomas, it is suggested to avoid circumferential irradiation or at least to spare a cutaneous strip of ≥1 cm to allow lymphatic drainage. However, such a recommendation is valid for the characteristic high doses (up to and over 70 Gy) used for a curative purpose in sarcoma treatment, which also consists of surgery that could disrupt the physiological lymph drainage. Firstly, lymphatic vessels are considered radio-resistant, and their damage is mediated by an extrinsic compression due to a radiation-induced fibrosis in the surrounding tissues. It is reasonable to assume that higher doses enhance the risk of developing dense fibrous tissue that blocks lymphatic flow. This phenomenon can be further increased by contiguous doses in boundary regions at risk of overlapping fields [19]. After all, the common daily clinical practice proves that palliative doses, such as that used in our case, even in other scenarios seem to be unable to produce skin fibrosis. Secondly, lymph nodes are highly radiosensitive and potentially damaged by lower doses that are enough to affect the ability to filter afferent lymphatic flow. For such reasons, the use of the PROSTEP™ device was useful not only for better patient comfort, by relaxing her back and ensuring the reproducibility of the target position, but also because bending the knee to approximately 120° allows us to more easily spare the popliteal lymph nodes: indeed, if we consider that the dose delivery was conducted with constant source-to-axis distance (SAD), the knee bending distances the popliteal fossa from the radiation source and so reduces photon beam energy deposition in the nearby lymph nodes. Such a sparing was further maximized by the delineation of an ad hoc avoidance structure behind the knee. On the opposite kneecap side, we were able to tailor a satisfying field junction by contouring a horseshoe-shaped area, thus forcing our treatment planning system (TPS) to compute a homogeneous dose coverage even at this level, thanks to the potentiality of the inverse planning method [14,15]. This latter approach, in fact, is much more versatile and less cumbersome than that proposed by Wooden et al. [20], who achieved the same result (irradiation of a circumferential superficial target) by using a six-field electron technique. In comparison, our solution is characterized by a more homogeneous dose distribution as well as a much better dose conformity, which results in a significant lower dose to bones (femur and tibia). Regarding bone sparing, our achievement should decrease the risk for radiation-related pathological fracture [21]. In such an effort, we reached a femur mean dose that is about half of the target prescription dose. Just as Steven et al. [8] concluded, IMRT techniques, by virtue of a greater dose conformity, are potentially able to reduce toxicity to OARs but not to improve survival outcomes [22]. These techniques have already been effectively tested in other clinical scenarios demanding a significant sparing of some OARs, e.g., spinal cord or urethra, similarly located within a surrounding radiotherapy target [23,24,25], or for reirradiation [26]. We know that other radiotherapy techniques are equally effective in the treatment of cutaneous targets [27,28,29,30], but their use is less suitable for highly complex clinical situations such as the present one. Our work presents a similar arrangement to the that used by Servy et al. [31], but with a more challenging circumferential target around a very close organ-at-risk that needs to be spared. As compared to that study, our therapeutic goals were partially achieved: (1) we observed a complete lasting regression of all skin nodules, except for that which developed three months after treatment in the intentionally underdosed popliteal fossa; (2) we were not able to avoid a worsening of lymphedema, which likely occurred because of metastases in lymph nodes proximal to the PTV. However, we are unable to assess what the burden is of such an extended circumferential field irradiation on the worsening of lymphedema. On the other hand, the effectiveness of our approach in stopping the progression of skin nodules is particularly remarkable. In fact, we must not leave out that, in some cases involving the extremities, cancer disease may be so severe and extensive that amputation is unavoidable [32]. Interestingly, the only new nodule arose in an unirradiated site, which was encompassed by a low scattered dose. This nodule was asymptomatic until patient death. Our case presentation is similar to that described by Blumenthal et al. but differs from it for bone managing (VMAT vs. IMRT/electron beam RT), dose prescription (46/54 Gy vs. 30/40 Gy) and follow-up time (2 months vs. 7 months) [33]. Due to the limited survival reported in our case, the technical approach by VMAT might have been useless and essentially equivalent with respect to less efficient techniques in these clinical scenarios. However, we think that this technique could be a valuable option in treating other extremity cancers with a more favourable prognosis, i.e., Kaposi’s sarcoma [34,35]. Skin represents a continuous matter of debate among radiation oncologists and other health professionals, even for sentinel events [36]. Even if the findings of this case report could be ultimately considered as negative from a merely prognostic point of view, they call for large clinical trials in order to identify any role for VMAT capabilities in the clinical scenario here investigated. Indeed, the achievement of an effective local control on lower extremity skin without the appearance of new in-field lesions deserves further investigations, even if only in a palliative context. This very preliminary report has the merit of informing the insiders about the possibility of adequately treating such challenging targets while sparing OARs thanks to the advances in radiotherapy techniques.

## 4. Conclusions

The usefulness of high-performance VMAT in treating patients with several in-transit MCC metastases to the entire skin of the lower extremities is questionable. MCC nodules are highly radiosensitive, and low palliative doses could be sufficient for local control in metastatic settings. Appropriate skin care in metastatic MCC patients is fundamental for preventing complications in skin lesions. Among such patients, lymphedema is still a critical issue: how and if radiotherapy may be implemented for this purpose is not yet clear. The issue needs further investigation, bearing in mind the potential deleterious impact of radiation on the lymph system. VMAT capabilities could be largely beneficial in treating other extremity cancers, even ulcerating ones.

### Additional Considerations: Weaknesses and Strengths

Since this is a case report, no definitive conclusion can be drawn regarding the usefulness of such a very large extended field RT in preventing the progression of MCC nodules along lower-extremity skin: an adequate sample size is needed to confirm this isolated case. Moreover, even though in this specific case it may be reasonably assumed that the worsening of lymphedema was due to the proximal off-target disease progression, a larger study could further substantiate or disprove this finding by clarifying the effect of a low circumferential radiation dose on lower limb lymphatic drainage. In the present case, some small skin areas received a “double” treatment for the sum of the previous RT courses (30 Gy in 10 fractions) with the last one (30 Gy in 15 fractions followed by a 10 Gy boost for clinical lesions), since we had in mind the good skin tolerance to high radiation doses [37,38]. However, we are unable to indicate a safety threshold in terms of the maximum extent possible for the field overlap of multiple palliative RT treatments. The limited patient survival hampered an assessment of any lasting benefit from VMAT use.

Strong points of this experience are: the reproducibility of the method; the ability to satisfactorily irradiate such a large and superficial target without a bolus, whose use might create an inconstant air gap over skin that would be enough to alter dose distribution; the easy accessibility of this technique compared to equally high-performing but less widespread alternatives such as surface-mold computer-optimized high-dose-rate brachytherapy [11,39].

Last but not least, even if this VMAT application failed to prolong patient survival, it achieved an effective stop of nodular disease progression towards dramatic complications, maintaining an acceptable quality of life [40].

## Data Availability

The data that support the findings of this study are available on request from the authors.

## References

[1] Singh G.K., Sinha A., Mishra P.S., Jain A., Beniwal N.S. (2021). Ulcerative Variant of Merkel Cell Carcinoma in an Immunocompetent Individual: An Unusual Presentation. Indian Dermatol. Online J..

[2] Gaba S., Chopra P., Pankaj P., Belho E.S., Qadri A.B., Aggarwal S. (2012). Merkel cell carcinoma—a rare cause of non-healing skin ulcer: A case report. J. Indian Med. Assoc..

[3] Shinogi T., Nagase K., Inoue T., Sato K., Onita A., Takamori A., Narisawa Y. (2021). Merkel cell carcinoma: A systematic review of the demographic and clinical characteristics of 847 cases in Japan. J. Dermatol..

[4] Carter J., Huang H.Q., Armer J., Carlson J.W., Lockwood S., Nolte S., Kauderer J., Hutson A., Walker J.L., Fleury A.C. (2021). GOG 244—The Lymphedema and Gynecologic cancer (LeG) study: The impact of lower-extremity lymphedema on quality of life, psychological adjustment, physical disability, and function. Gynecol. Oncol..

[5] Rodrigues M.A., Caetano M., Amorim I., Selores M. (2021). Dermo-Hipodermites Bacterianas Agudas Não Necrotizantes: Erisipela e Celulite Infeciosa [Non-Necrotizing Acute Dermo-Hypodermal Infections: Erysipela and Infectious Cellulitis]. Acta Med. Port..

[6] Barreira J.V., Valejo Coelho M.M., Ribeiro C., Semedo M. (2019). Unknown primary Merkel cell carcinoma with cutaneous spread. BMJ Case Rep..

[7] Poulsen M., Round C., Keller J., Tripcony L., Veness M. (2010). Factors influencing relapse-free survival in Merkel cell carcinoma of the lower limb—a review of 60 cases. Int. J. Radiat. Oncol. Biol. Phys..

[8] Steven N., Lawton P., Poulsen M. (2019). Merkel Cell Carcinoma—Current Controversies and Future Directions. Clin. Oncol..

[9] Zerini D., Patti F., Spada F., Fazio N., Pisa E., Pennacchioli E., Prestianni P., Cambria R., Pepa M., Grana C.M. (2021). Multidisciplinary team approach for Merkel cell carcinoma: The European Institute of Oncology experience with focus on radiotherapy. Tumori J..

[10] Pacella J., Ashby M., Ainslie J., Minty C. (1988). The role of radiotherapy in the management of primary cutaneous neuroendocrine tumors (Merkel cell or trabecular carcinoma): Experience at the Peter MacCallum Cancer Institute (Melbourne, Australia). Int. J. Radiat. Oncol. Biol. Phys..

[11] Garibyan L., Cotter S.E., Hansen J.L., Noell C., Dorosario A., O’Farrell D.A., Devlin P.M., Wang L.C. (2013). Palliative treatment for in-transit cutaneous metastases of Merkel cell carcinoma using surface-mold computer-optimized high-dose-rate brachytherapy. Cancer J..

[12] Veness M., Howle J. (2010). Patients with clinically node negative extremity Merkel cell carcinoma: The importance of identifying and treating patients with microscopic nodal metastases. Australas. J. Dermatol..

[13] Semwal M.K. (2020). Khan’s The Physics of Radiation Therapy. J. Med. Phys..

[14] Fogliata A., Bergström S., Cafaro I., Clivio A., Cozzi L., Dipasquale G., Hållström P., Mancosu P., Navarria P., Nicolini G. (2011). Cranio-spinal irradiation with volumetric modulated arc therapy: A multi-institutional treatment experience. Radiother. Oncol..

[15] Maddalo M., Benecchi G., Altabella L., Ghetti C., D’Abbiero N. (2021). Automatic feathering algorithm for VMAT craniospinal irradiation: A comprehensive comparison with other VMAT planning strategies. Med Dosim..

[16] Executive Committee of the International Society of Lymphology (2020). The diagnosis and treatment of peripheral lymphedema: 2020 Consensus Document of the International Society of Lymphology. Lymphology.

[17] Ferini G., Valenti V., Tripoli A., Illari S.I., Molino L., Parisi S., Cacciola A., Lillo S., Giuffrida D., Pergolizzi S. (2021). Lattice or Oxygen-Guided Radiotherapy: What If They Converge? Possible Future Directions in the Era of Immunotherapy. Cancers.

[18] Allam O., Park K.E., Chandler L., Mozaffari M.A., Ahmad M., Lu X., Alperovich M. (2020). The impact of radiation on lymphedema: A review of the literature. Gland. Surg..

[19] Hayes S.B., Freedman G.M., Li T., Anderson P.R., Ross E. (2008). Does axillary boost increase lymphedema compared with supraclavicular radiation alone after breast conservation?. Int. J. Radiat. Oncol. Biol. Phys..

[20] Wooden K.K., Hogstrom K.R., Blum P., Gastorf R.J., Cox J.D. (1996). Whole-limb irradiation of the lower calf using a six-field electron technique. Med Dosim. Off. J. Am. Assoc. Med Dosim..

[21] Soares C.B.G., De Araújo I.D., Pádua B.J., Vilela J.C.S., Souza R.H.R., Teixeira L.E.M. (2019). Pathological fracture after radiotherapy: Systematic review of literature. Rev. Assoc. Med. Bras..

[22] Ferini G., Tripoli A., Umina V., Borzì G.R., Marchese V.A., Illari S.I., Cacciola A., Lillo S., Parisi S., Valenti V. (2021). Radiation Proctitis: The Potential Role of Hyaluronic Acid in the Prevention and Restoration of Any Damage to the Rectal Mucosa among Prostate Cancer Patients Submitted to Curative External Beam Radiotherapy. Gastroenterol. Insights.

[23] Mallory M., Pokhrel D., Badkul R., Jiang H., Lominska C., Wang F. (2018). Volumetric modulated arc therapy treatment planning of thoracic vertebral metastases using stereotactic body radiotherapy. J. Appl. Clin. Med Phys..

[24] Pontoriero A., Iatì G., Cacciola A., Conti A., Brogna A., Siragusa C., Ferini G., Davì V., Tamburella C., Molino L. (2020). Stereotactic Body Radiation Therapy With Simultaneous Integrated Boost in Patients With Spinal Metastases. Technol. Cancer Res. Treat..

[25] Jaccard M., Zilli T., Dubouloz A., Escude L., Jorcano S., Linthout N., Bral S., Verbakel W., Bruynzeel A., Björkqvist M. (2020). Urethra-Sparing Stereotactic Body Radiation Therapy for Prostate Cancer: Quality Assurance of a Randomized Phase 2 Trial. Int. J. Radiat. Oncol. Biol. Phys..

[26] Vadalà R.E., Santacaterina A., Sindoni A., Platania A., Arcudi A., Ferini G., Mazzei M.M., Marletta D., Rifatto C., Risoleti E.V.I. (2016). Stereotactic body radiotherapy in non-operable lung cancer patients. Clin. Transl. Oncol. Off. Publ. Fed. Span. Oncol. Soc. Natl. Cancer Inst. Mex..

[27] Ferini G., Molino L., Bottalico L., De Lucia P., Garofalo F. (2021). A small case series about safety and effectiveness of a hypofractionated electron beam radiotherapy schedule in five fractions for facial non melanoma skin cancer among frail and elderly patients. Rep. Pract. Oncol. Radiother..

[28] Pontoriero A., Iatì G., Pergolizzi S. (2015). A case report of a patient with squamous cell carcinoma of the face irradiated using a stereotactic technique. Radiat. Oncol. J..

[29] Coles A. (2010). A patient presenting with an advanced squamous cell carcinomas to the left thigh. Radiographer.

[30] Fitzgerald E., Miles W., Fenton P., Frantzis J. (2014). Intensity-modulated radiation therapy to bilateral lower limb extremities concurrently: A planning case study. J. Med Radiat. Sci..

[31] Servy A., Kramkimel N., Franck N., Park S., Kirova Y. (2014). Helical tomotherapy in oncodermatology: Case report of circumferential cutaneous lymphoma treated by this optimized radiotherapy. Cancer Radiother. J. Soc. Fr. Radiother. Oncol..

[32] Gunaratne D.A., Howle J.R., Veness M.J. (2016). Merkel cell carcinoma: A case of palliative upper limb amputation in a patient with refractory in-transit metastases. Australas. J. Dermatol..

[33] Blumenthal L., VandenBoom T., Melian E., Peterson A., Hutchens K.A. (2015). Multiple Primary Merkel Cell Carcinomas Presenting as Pruritic, Painful Lower Leg Tumors. Case Rep. Dermatol..

[34] Pergolizzi S., Santacaterina A., Gaeta M., Blandino A. (2009). Kaposi’s sarcoma in young patients treated with orthovoltage irradiation and having a minimum follow-up of forty-six years. Tumori.

[35] Narayan J. (2012). Treatment options for Classic Kaposi’s. Radiographer.

[36] Sindoni A., Severo C., Vadala’ R.E., Ferini G., Mazzei M.M., Vaccaro M., Iatì G., Pontoriero A., Pergolizzi S. (2016). Levetiracetam-induced radiation recall dermatitis in a patient undergoing stereotactic radiotherapy. J. Dermatol..

[37] Ferini G., Molino L., Tripoli A., Valenti V., Illari S.I., Marchese V.A., Cravagno I.R., Borzi G.R. (2021). Anatomical Predictors of Dosimetric Advantages for Deep-inspiration-breath-hold 3D-conformal Radiotherapy Among Women With Left Breast Cancer. Anticancer Res..

[38] Iatì G., Parisi S., Santacaterina A., Pontoriero A., Cacciola A., Brogna A., Platania A., Palazzolo C., Cambareri D., Davì V. (2020). Simultaneous Integrated Boost Radiotherapy in Unresectable Stage IV (M0) Head and Neck Squamous Cell Cancer Patients: Daily Clinical Practice. Rep. Pract. Oncol. Radiother..

[39] Tagliaferri L., Ciardo F.G., Fionda B., Casà C., DIStefani A., Lancellotta V., Placidi E., Macchia G., Capocchiano N.D., Morganti A.G. (2021). Non-melanoma Skin Cancer Treated by Contact High-dose-rate Radiotherapy (Brachytherapy): A Mono-institutional Series and Literature Review. In Vivo.

[40] Stachyra K., Dudzisz-Śledź M., Bylina E., Szumera-Ciećkiewicz A., Spałek M.J., Bartnik E., Rutkowski P., Czarnecka A.M. (2021). Merkel Cell Carcinoma from Molecular Pathology to Novel Therapies. Int. J. Mol. Sci..

